# Veno-arterial extracorporeal membrane oxygenation in addition to primary PCI in patients presenting with ST-elevation myocardial infarction

**DOI:** 10.1007/s12471-017-1068-y

**Published:** 2017-12-19

**Authors:** F. S. van den Brink, A. D. Magan, P. G. Noordzij, C. Zivelonghi, P. Agostoni, F. D. Eefting, J. M. ten Berg, M. J. Suttorp, B. R. Rensing, J. P. van Kuijk, P. Klein, E. Scholten, J. A. S. van der Heyden

**Affiliations:** 1 Department of Cardiology, St Antonius Hospital, Nieuwegein, The Netherlands; 2 Department of Intensive Care, St Antonius Hospital, Nieuwegein, The Netherlands; 3 Department of Cardio-Thoracic Surgery, St Antonius Hospital, Nieuwegein, The Netherlands

**Keywords:** ECMO, STEMI, Cardiogenic Shock, Circulatory Support

## Abstract

**Introduction:**

Primary percutaneous coronary intervention (pPCI) in ST-elevation myocardial infarction (STEMI) can cause great haemodynamic instability. Veno-arterial extracorporeal membrane oxygenation (VA-ECMO) can provide haemodynamic support in patients with STEMI but data on outcome and complications are scarce.

**Methods:**

An in-hospital registry was conducted enrolling all patients receiving VA-ECMO. Patients were analysed for medical history, mortality, neurological outcome, complications and coronary artery disease.

**Results:**

Between 2011 and 2016, 12 patients underwent pPCI for STEMI and received VA-ECMO for haemodynamic support. The majority of the patients were male (10/12) with a median age of 63 (47–75) years and 4 of the 12 patients had a history of coronary artery disease. A cardiac arrest was witnessed in 11 patients. The left coronary artery was compromised in 8 patients and 4 had right coronary artery disease. All patients were in Killip class IV. Survival to discharge was 67% (8/12), 1‑year survival was 42% (5/12), 2 patients have not yet reached the 1‑year survival point but are still alive and 1 patient died within a year after discharge. All-cause mortality was 42% (5/12) of which mortality on ECMO was 33% (4/12). Patient-related complications occurred in 6 of the 12 patients: 1 patient suffered major neurological impairment, 2 patients suffered haemorrhage at the cannula site, 2 patients had limb ischaemia and 1 patient had a haemorrhage elsewhere. There were no VA-ECMO hardware malfunctions.

**Conclusion:**

VA-ECMO in pPCI for STEMI has a high survival rate and neurological outcome is good, even when the patient is admitted with a cardiac arrest.

## Introduction

Primary percutaneous coronary intervention (pPCI) is the cornerstone in the treatment of ST-elevation myocardial infarction (STEMI). STEMI can cause great haemodynamic instability through a mechanism of cardiac failure and subsequent low output state [1–7]. Haemodynamic support following STEMI is quintessential for survival and preservation of cardiac function [6]. Historically, this consisted of medical support with inotropes and vasopressors, and mechanical circulatory support by an intra-aortic balloon pump [1, 3, 8, 9]. Recently more advanced devices have been introduced such as the Impella device, but data on its safety and efficacy are inconclusive [10, 11].

Another form of haemodynamic support is veno-arterial extracorporeal membrane oxygenation (VA-ECMO) [12, 13]. VA-ECMO can provide haemodynamic support in patients with STEMI with cardiac failure and possible concomitant respiratory failure due to pulmonary congestion [13–15]. Its mechanism is based on the combined possibility of circulatory support through laminar flow of blood in the aorta and simultaneous oxygenation of the patient’s blood. Blood flow of up to 7 litres/min can be generated in ideal circumstances.

Data on outcome and complications in the use of VA-ECMO in pPCI for STEMI are scarce but first reports on this therapy show promising results [6, 14–16]. This study was performed to gain insight into the outcome and complications of the use of VA-ECMO in pPCI for STEMI with subsequent haemodynamic instability.

## Methods

An in-hospital registry was kept at the St Antonius Hospital, Nieuwegein, the Netherlands involving all patients who received treatment with VA-ECMO for haemodynamic support from 2009 onwards. Data were collected retrospectively and included those patients who received VA-ECMO in addition to pPCI for STEMI. All patients were analysed at baseline for age, sex, medical history, previous coronary artery disease, coronary artery occlusion in STEMI, concomitant coronary artery disease, SYNTAX score, concomitant chronic total occlusion, Survival After VA-ECMO (SAVE) score, procedural characteristics including mode of cannulation and concomitant use of other circulatory support (e. g. intra-aortic balloon pump (IABP)), left ventricular function prior to admission, survival, complications related to the patient (e. g. bleeding at the cannula site, limb ischaemia), complications related to the ECMO hardware (e. g. pump failure, clot formation), length of ECMO treatment and length of stay on the ICU and in hospital, haemodynamic parameters, mortality and neurological outcome [17–19].

Shock was defined according to the Killip class [20]. The SYNTAX score was calculated using the online SYNTAX score calculator (http://www.syntaxscore.com/calculator/start.htm) [17]. The SAVE score, an Extracorporeal Life Support Organisation (ELSO) endorsed and validated score, was calculated to compare predicted mortality with actual morality [18]. This was done by using the online SAVE score calculator (http://www.save-score.com/). Neurological outcome was defined using the Cerebral Performance Categories Scale (CPC) where a CPC score of 1 and 2 was deemed a good neurological outcome [19].

## Results

VA-ECMO using the Maquet Cardiohelp, which is a system using an integrated rotational pump and oxygenator, was introduced at the St Antonius Hospital in Nieuwegein, the Netherlands, a large referral centre, in 2009. From 2009 until 2016 a total of 118 patients received VA-ECMO for circulatory support for various conditions such as post cardiotomy, pulmonary embolism and septic shock. Between 2009 and 2016 a total of 19,116 patients underwent coronary intervention of which 3,673 underwent a PCI for an acute ischaemic event; 12 patients underwent pPCI for STEMI and received additional VA-ECMO treatment for haemodynamic support. The first VA-ECMO in addition to STEMI was performed in 2011.

### Baseline characteristics

The majority of patients were male (83%, 10/12) with a median age of 63 (47–75) years with only 1 patient over the age of 70 years. A history of coronary artery disease in the form of a previous PCI was present in 33% (4/12) of the patients of whom 1 patient had recently diagnosed left main disease. None of the patients had undergone previous cardiac surgery and none had known left ventricular impairment prior to admission. None of the patients had a history of neurological events in the form of either a transient ischaemic attack or a cerebrovascular accident. Only 1 patient had known pulmonary disease in the form of chronic obstructive pulmonary disease GOLD class 1/4. Two patients had a history of diabetes mellitus type 2 and 42% (5/10) were known to have a history of hypertension. There was 1 patient with a history of peripheral artery disease in the form of implantation of an aortic bifurcation prosthesis 11 years prior to primary pPCI for STEMI. All patients were in cardiogenic shock, Killip class 4 prior to and after pPCI (Table 1).

**Table 1:** Baseline characteristics

Baseline characteristics	*N* = 12
Male	83% (10/12)
Age	63 (47–75) years
Previous coronary artery disease	33% (4/12)
Diabetes	17% (2/12)
Hypertension	42% (5/12)
TIA/Stroke	NA
COPD	8% (1/12)
Per peripheral artery disease	8% (1/12)
Shock upon admission, Killip class IV	100% (12/12)

*TIA* transient ischaemic attack, *COPD* chronic obstructive pulmonary disease, *NA* not available

### Procedural characteristics

The indication for VA-ECMO was determined by the attending interventional cardiologist and intensivist. An out-of-hospital cardiac arrest (OHCA) prior to presentation was witnessed in 9 of the 12 patients. Two patients suffered an in-hospital cardiac arrest. The left coronary artery was involved in 67% (8/12) of the patients: 3 left main coronary arteries, 5 left anterior descending arteries and the other 4 patients had right coronary artery disease. The mean SYNTAX score was 23.7 (4–40). A total of 8 patients had concomitant coronary artery disease, 3 of which had a chronic total occlusion of another coronary artery than the culprit lesion. Culprit lesion revascularisation was achieved in 92% (11/12). One patient had an unsuccessful evacuation of thrombus in the right coronary artery. One patient had successful revascularisation of the culprit lesion but received a coronary artery bypass graft (CABG) after pPCI.

Mean systolic blood pressure was 70 (53–80) mm Hg upon admission while mean pH was 7.2 (6.97–7.40) and mean lactate was 6.4 (1.6–12.0) mmol/l. Mean blood bicarbonate levels were 16.5 (5.7–22.2) mmol/l and mean creatinine was 134 (72–333) mmol/l.

All patients were cannulated at the Cathlab. The cannula was inserted in the femoral artery in 9 of the 12 patients. In 3 patients the cannula was placed in the subclavian artery by the attending cardiothoracic surgeon. The venous cannula was placed in the femoral vein in all but 1 patient, where it was placed in the internal jugular vein. Two patients had an IABP in situ at the time of cannulation. The mean SAVE score was −5.5 (−2–−11) which represents an estimated survival of 30% (25–35%) (Table 2).

**Table 2:** Procedural characteristics

Procedural characteristics	*N* = 12
Out-of-hospital cardiac arrest	75% (9/12)
In-hospital cardiac arrest	17% (2/12)
Left coronary artery	67% (8/12)
Right coronary artery	33% (4/12)
Culprit revascularisation	92% (11/12)
SYNTAX score	23.7 (4–40)
Intra-aortic balloon pump	17% (2/12)
Subsequent CABG after PCI	8% (1/12)
SAVE score	5.5 (−2–−11)

*CABG* coronary artery bypass grafting, *PCI* percutaneous coronary intervention

Initially the patients were weaned off the device according to the clinical judgement of the attending physician. However, after 2015 the patients were weaned off the device using the local weaning protocol, which is a modification of an existing protocol [21].

### Outcome

Survival to discharge was 67% (8/12), 1‑year survival was 42% (5/12), and 2 of the 12 patients have not yet reached the 1‑year survival point but are still alive. Mortality on VA-ECMO was 33% (4/12) and this was also the 30-day mortality. All patients who survived VA-ECMO survived to discharge. All-cause mortality was 42% (5/12); in the aforementioned 33% (4/12) of patients who died on VA-ECMO and 1 patient who died within a year after discharge due to aspiration pneumonia, this was caused by poor neurological status after prolonged cardiopulmonary resuscitation.

Both the patients who received IABP and ECMO survived to discharge. The patient who suffered unsuccessful evacuation of the thrombus in the right coronary artery did not survive. The patient who underwent subsequent CABG did survive to discharge.

Patient-related complications occurred in 6 of the 12 patients: 2 patients suffered haemorrhage at the cannula site and 1 patient had a haemorrhage elsewhere in the form of a venous haemorrhage of the liver due to the venous cannula position while on VA-ECMO. Limb ischaemia occurred in 3 of the 12 patients; in 1 of these patients the right leg was eventually amputated due to clot formation between the ECMO cannula and antegrade leg perfusion. One patient suffered major neurological impairment; however, this was not due to intracranial haemorrhage but due to prolonged resuscitation prior to receiving the VA-ECMO treatment. There were no malfunctions regarding the VA-ECMO hardware. The median time spent on VA-ECMO was 5 (1–10) days, the median time spent on the ICU was 14 (1–68) days and the median time spent in hospital was 23 (1–82) days *(*Table 3).

**Table 3:** Outcome

Outcome	*N* = 12
Survival to discharge	67% (8/12)
Survival with >1 year follow-up	42% (5/12)
All-cause mortality	42% (5/12)
Mortality on ECMO	33% (4/12)
30-day mortality	33% (4/12)
Mortality after discharge	8% (1/12)
All complications	50% (6/12)
Major neurological impairment	8% (1/12)
Haemorrhage cannula	17% (2/12)
Haemorrhage elsewhere	8% (1/12)
Limb ischaemia	25% (3/12)
Median time on ECMO	5 (1–10) days
Median time on ICU	14 (1–82) days

*ECMO* extracorporeal membrane oxygenation; *ICU* intensive care unit

### Follow-up after VA-ECMO in pPCI for STEMI

Of the 12 patients who received VA-ECMO in pPCI for STEMI, 1 patient received a CABG for concomitant coronary artery disease. Another patient underwent implantation of a left ventricular assist device (LVAD) in a designated LVAD centre. Both of these patients survived to discharge and are neurologically well.

## Discussion

This study shows that VA-ECMO is feasible in selected patients. It could potentially improve survival in patients undergoing primary PCI for STEMI with cardiogenic shock. As all patients were in cardiogenic shock, VA-ECMO could be considered a last resort therapy. There are no randomised trials on VA-ECMO in pPCI in STEMI; observational studies proving the concept of circulatory support with VA-ECMO in pPCI in STEMI are crucial in facilitating future research in this field [13–16]. In this observational study VA-ECMO was feasible, safe and with a relatively low complication rate in a very high-risk group of patients with great haemodynamic instability. As our research shows, the applicability of this possibly life-saving technique in patients undergoing pPCI for STEMI could be the start of a future randomised controlled trial, which may prove its use in pPCI in STEMI.

Although the SAVE score is not a substitute for clinical assessment of patients, the difference between the predicted mortality and the actual mortality may illustrate that VA-ECMO is especially useful in the treatment of cardiogenic shock caused by STEMI.

Only 1 patient suffered a poor neurological outcome due to prolonged resuscitation prior to pPCI and VA-ECMO cannulation. This illustrates the safety of using VA-ECMO in haemodynamic support with respect to possible neurological injury. Three patients suffered limb ischaemia. This is a known complication of VA-ECMO due to occlusion of the femoral artery distal of the cannula and the cannula itself. The correct use of antegrade leg perfusion via insertion of an extra small diameter cannula can prevent this (known as leg-ECMO or L‑ECMO). However, 1 patient had adequate leg perfusion but developed clot formation between the retrograde ECMO cannula and antegrade leg perfusion. Surgical exploration during decannulation could prevent this. Studies in this regard are lacking and a standardised procedure of decannulation might prevent this complication. No intracranial haemorrhages were reported, although patients on VA-ECMO received dual antiplatelet therapy and heparin infusion for the ECMO circuit. This indicates the safety of the described procedure from a neurological point of view.

One of the possible drawbacks of VA-ECMO in pPCI for STEMI is the increase in afterload of the heart. This is a direct result of competitive flow of the ECMO circuit with the cardiac circulation [22]. However, as one of the major problems of cardiogenic shock is poor organ perfusion with subsequent organ failure, adequate organ perfusion is pivotal in improving patient survival and may outweigh the problem of increased afterload. In our study increased afterload did not lead to a worsening of the outcome for patients. If increasing afterload poses a problem in the treatment of patients on VA-ECMO, the use of an IABP or Impella device for left ventricular unloading may be considered. However, the possible benefit of left ventricular unloading is purely theoretical and not yet proven in a clinical setting. Furthermore, the use of an IABP or Impella or other left ventricular unloading device may increase the risk of possible complications, the complexity of the procedure as well as the cost of treatment. In this study, 2 patients received concomitant IABP therapy during VA-ECMO treatment and survived to discharge. This illustrates the technical feasibility of these two combined techniques.

Two of the described patients underwent VA-ECMO treatment in pPCI for STEMI and received additional therapy after pPCI in the form of a CABG and LVAD placement. This underlines the possibility of using VA-ECMO as a bridge to LVAD, treatment or recovery.

In this study, all patients were placed on VA-ECMO after PCI. In future studies patients could be placed on VA-ECMO prior to PCI. The first strategy has the benefit of earlier revascularisation, but it prolongs poor organ perfusion and haemodynamic shock. The second strategy may delay revascularisation by several minutes but allows early haemodynamic support and stabilisation as VA-ECMO therapy can be administered directly in the Cathlab. Future research must establish the ideal timing of VA-ECMO placement.

The absence of ECMO-related hardware failure is a possible indication of the reliability of the Maquet Cardiohelp. It can be safely used in this group of patients.

### Limitations

Our study has several limitations. It is an observational study comprising only a small number of patients. As patients were selected for VA-ECMO by the attending physicians, selection bias is present. Furthermore, there was no control group; future studies may be able to randomise patients undergoing pPCI for STEMI with subsequent cardiogenic shock to VA-ECMO treatment versus medical treatment. The small number of patients in this study makes any sound statistical conclusion impossible. In future registries with larger numbers of patients, this can be overcome.

## Conclusion

In our group, VA-ECMO in pPCI for STEMI had a survival to discharge rate of 67% and even when patients were admitted with an OHCA this outcome was favourable. The complication rate was relatively low and neurological outcome was good. Further research is needed to identify the patients most likely to benefit from VA-ECMO treatment in pPCI for STEMI. Randomised controlled trials are needed to prove the efficacy of this new and promising technique.
